# Supplementary material for: Distinct populations of cortical pyramidal neurons mediate drug reward and aversion
Source: Nat Commun. 2021 Jan 8;12:182. doi: 10.1038/s41467-020-20526-0 (PMC7794564; doi:10.1038/s41467-020-20526-0)
Supplement: Supplementary file 2 — Reporting Summary [file 41467_2020_20526_MOESM2_ESM.pdf]

nature research

Corresponding author(s): Susan Ferguson

Last updated by author(s): Nov 8, 2020

Reporting Summary

Nature Research wishes to improve the reproducibility of the work that we publish. This form provides structure for consistency and transparency in reporting. For further information on Nature Research policies, see our Editorial Policies and the Editorial Policy Checklist.

Statistics

For all statistical analyses, confirm that the following items are present in the figure legend, table legend, main text, or Methods section.

n/a	Confirmed	
☐	☒	The exact sample size (n) for each experimental group/condition, given as a discrete number and unit of measurement
☐	☒	A statement on whether measurements were taken from distinct samples or whether the same sample was measured repeatedly
☐	☒	The statistical test(s) used AND whether they are one- or two-sided<br>*Only common tests should be described solely by name; describe more complex techniques in the Methods section.*
☐	☒	A description of all covariates tested
☐	☒	A description of any assumptions or corrections, such as tests of normality and adjustment for multiple comparisons
☐	☒	A full description of the statistical parameters including central tendency (e.g. means) or other basic estimates (e.g. regression coefficient) AND variation (e.g. standard deviation) or associated estimates of uncertainty (e.g. confidence intervals)
☐	☒	For null hypothesis testing, the test statistic (e.g. F, t, r) with confidence intervals, effect sizes, degrees of freedom and P value noted<br>*Give P values as exact values whenever suitable.*
☒	☐	For Bayesian analysis, information on the choice of priors and Markov chain Monte Carlo settings
☒	☐	For hierarchical and complex designs, identification of the appropriate level for tests and full reporting of outcomes
☒	☐	Estimates of effect sizes (e.g. Cohen's d, Pearson's r), indicating how they were calculated

Our web collection on statistics for biologists contains articles on many of the points above.

Software and code

Policy information about availability of computer code

Data collection	ELP USB camera and VLC media player
Data analysis	Microsoft Excel (Version 16), Prism (Version 8), ImageJ (Version 1.52s)

For manuscripts utilizing custom algorithms or software that are central to the research but not yet described in published literature, software must be made available to editors and reviewers. We strongly encourage code deposition in a community repository (e.g. GitHub). See the Nature Research guidelines for submitting code & software for further information.

Data

Policy information about availability of data

All manuscripts must include a data availability statement. This statement should provide the following information, where applicable:

- Accession codes, unique identifiers, or web links for publicly available datasets
- A list of figures that have associated raw data
- A description of any restrictions on data availability

The data included in this paper are provided in a source data file.

Field-specific reporting

Life sciences study design

All studies must disclose on these points even when the disclosure is negative.

Sample size	The number of animals used in the present experiments were based on those used in similar publications.
Data exclusions	Nine rats were excluded from the experiments for lack of viral expression and one rat was excluded because he did not consume M&Ms.
Replication	All experiments were performed independently, except for the data in Figure 2C which was performed in multiple waves and replicated across waves. Control rats from independent experiments were compared to confirm replication of baseline behavior, all baseline behavior was replicated.
Randomization	Rats were randomly assigned to a group prior to the onset of each experiment.
Blinding	Investigators were blinded to group during data collection and analysis.

Reporting for specific materials, systems and methods

We require information from authors about some types of materials, experimental systems and methods used in many studies. Here, indicate whether each material, system or method listed is relevant to your study. If you are not sure if a list item applies to your research, read the appropriate section before selecting a response.

Materials & experimental systems

n/a	Involved in the study	
☐	☒	Antibodies
☒	☐	Eukaryotic cell lines
☒	☐	Palaeontology and archaeology
☐	☒	Animals and other organisms
☒	☐	Human research participants
☒	☐	Clinical data
☒	☐	Dual use research of concern

Methods

n/a	Involved in the study	
☒	☐	ChIP-seq
☒	☐	Flow cytometry
☒	☐	MRI-based neuroimaging

Antibodies

Antibodies used	Primary: rabbit anti-DS Red (Clontech, #632496), mouse anti-GFP (Millipore, #MAB3580), Secondary: goat anti-rabbit-Alex 568 (Invitrogen A-11036), goat anti-mouse Alexa 488 (Invitrogen, #A-11029)
Validation	All antibodies that were used are commercially available and widely used, so we do not validate the antibodies in house. For primary antibodies, the companies performed purity analysis using SDS-polyacrylamide gel electrophoresis with Coomaise Blue staining and purity and functioning was confirmed using absorption spectrum analysis. For the secondary antibodies, a certificate of analysis is provided for each lot, and it was stated on their websites that extensive antibody testing was performed in rat tissue.

Animals and other organisms

Policy information about studies involving animals; ARRIVE guidelines recommended for reporting animal research

Laboratory animals	Outbred male Sprague Dawley rats weighing 250-274 g (~12 weeks) upon arrival were obtained from Envigo.
Wild animals	Study did not involve wild animals.
Field-collected samples	Study did not involve samples collected from the field.
Ethics oversight	Experiments were performed according to NIH guidelines and were approved by the Seattle Children's Research Institute Institutional Animal Care and Use Committee.

Note that full information on the approval of the study protocol must also be provided in the manuscript.
